# Canine Parvovirus and Vaccine-Origin Feline Panleukopenia Virus in Wastewater, Arizona, USA: July 2022–June 2023

**DOI:** 10.3390/microorganisms13092124

**Published:** 2025-09-11

**Authors:** Jacqueline Vargas, Brenda Bermudez-Rivera, Izabella Block, Gray Shaffer, Lesley Estrada, Tegan Dadd, Tanner Dickerson, Courtney Curtis, Craig Woods, Erin M. Driver, Rolf U. Halden, Arvind Varsani, Matthew Scotch, Temitope O. C. Faleye

**Affiliations:** 1 Biodesign Center for Environmental Health Engineering, Biodesign Institute, Arizona State University, Tempe, AZ 85287, USA; jvarga48@asu.edu (J.V.);; 2 School of Life Sciences, Arizona State University, Tempe, AZ 85287, USA; 3 Complex Adaptive Systems, Arizona State University, Scottsdale, AZ 85287, USA; 4 School of Sustainable Engineering and the Built Environment, Arizona State University, Tempe, AZ 85287, USA; 5 Biodesign Center for Fundamental and Applied Microbiomics, Center for Evolution and Medicine, School of Life Sciences, Arizona State University, Tempe, AZ 85287, USA; 6 College of Health Solutions, Arizona State University, Tempe, AZ 85287, USA

**Keywords:** canine parvovirus, vaccine-origin feline panleukopenia virus, long-read sequencing, wastewater surveillance, United States

## Abstract

Canine parvovirus (CPV) is a virus of veterinary health significance and a member of the *Parvoviridae* family. Despite its clinical significance and global distribution, surveillance is often limited to cases serious enough to result in veterinary visit and/or hospitalization, thereby limiting our understanding of its evolution and diversity. In this study, we coupled wastewater surveillance (WWS), long-range polymerase chain reaction (PCR) and long-read sequencing and demonstrate the utility of this approach for community-level monitoring of parvovirus diversity. We screened archived viral concentrates from wastewater (WW) collected monthly from July 2022 to June 2023 as part of a previous virus surveillance study from a population of ~500,000 people in Maricopa County, Arizona, USA. Using long-range PCR, the coding-complete sequences (~4.5 kb) were amplified as single contigs and sequenced on a long-read sequencer (MinION). Reads were trimmed, assembled, and contigs subjected to a bioinformatics workflow that includes phylogenetics, immuno-informatics and protein structure modelling. The ~4.5 kb amplicons were amplified from all the samples and sequenced. Twelve contigs (length: 4555 nt to 4675 nt: GC%: 35% to 36%) were assembled from 86,858 trimmed and size-selected reads (length 4400 nt–4900 nt) and all typed as parvoviruses. Overall, there were 11 CPV variants (2a, 2b and 2c) and 1 feline panleukopenia virus (FPV) variant. The FPV was 100% similar in the VP2 genomic region to the 1964 Johnson snow leopard strain present in the Felocell vaccine, suggesting recent shedding post-vaccination. For the CPVs, our analysis showed multiple amino acid substitutions in the VP2 and NS1 proteins, suggestive of host immune pressure and viral adaptation, respectively. The CPV variants clustered predominantly with North and South American variants, suggesting transboundary viral movement and multiple CPV-2c transmission chains seem evident. To the best of our knowledge, we here document the first detection of vaccine-origin FPV in WW. We show the presence of CPV-2a, 2b and 2c in the population sampled and provide evidence that suggests transmission of CPVs across the Americas. Our results also show that WWS coupled with long-range PCR and long-read sequencing is a feasible population-level complement to clinical case surveillance that also facilitates detection of vaccine-origin virus variants. The model we demonstrate here for tracking parvoviruses can also be easily extended to other DNA viruses of human and veterinary health significance.

## 1. Introduction

Canine parvovirus (CPV) is an enteric pathogen of animal health significance to both canines and felines [1]. They often cause severe disease with outcomes ranging from mild illness to death. Treatment can be costly (USD 1000–2000 in the U.S.) and survival varies widely (9–90%). Hence, euthanasia is a frequent outcome for severe cases. CPV belongs to the family *Parvoviridae*, and some other members of this family of significance to public health include parvovirus B19, human bocavirus and feline panleukopenia virus (FPV). The parvovirus virions are non-enveloped icosahedral capsids (~26 nm in diameter) displaying 60 copies of a single surface motif of the structural protein [2]. The genome is a linear, single-stranded DNA (~5 kb) that encodes about five proteins (NS1, NS2, VP1, VP2 and SAT) in two major open reading frames (ORFs). Virus typing for CPV is usually performed using the VP2 structural (capsid) protein and four variants (2, 2a, 2b and 2c) were assigned based on amino acid substitutions in residues 80–568 of the VP2 protein, which contain key antigenic sites (Table 1) [1].

A variant of FPV evolved into CPV, causing a panzootic in the 1970s, and has since become endemic globally [1]. Point-of-care antigen testing is the standard diagnostic method, with genomic surveillance limited to a subset of those cases serious enough to necessitate a visit to the veterinarian. As a result, there is limited genomic information on CPV genomes, especially from asymptomatic and/or subclinical cases. This limits our understanding of CPV evolutionary dynamics. To complement clinical case surveillance, in June 2022, we explored sampling dog feces from so-called ‘fecal bags’ collected from outdoor waste bins in Arizona, USA [3]. However, only 1.4% (1/73) of dog fecal samples screened had CPV [3]. This highlighted the need for a complementary surveillance system that is cheaper, faster and more efficient.

Wastewater (WW) surveillance of pathogens has been demonstrated to be a faster, cost-effective, highly efficient, population-level surveillance system that can also function at an early warning capacity [4,5,6]. Furthermore, studies [7,8,9,10,11] have documented the detection of animal viruses with non-enveloped icosahedral capsids transmitted via the fecal–oral route in WW samples. Considering CPV fits this profile and our previous study [9] in the community sampled in this study had shown in WW the presence of viruses documented in multiple countries to be circulating in canines, we explored WW surveillance for CPV. However, the evolutionary rate (~10^−4^ substitutions/site/year) [12,13] of parvovirus DNA genomes and the need to monitor coevolution across multiple sites in the protein coding region (e.g., in the VP2 for typing, ~500 aa, ~1500 nt; Table 1) necessitated that we implement an approach that ensures we can recover coding-complete sequence as a single contig, and sequence the contigs using long-read technologies so that we can detect coevolving sites in the genome that are far apart while ruling out artificial chimeras. As a result, in this study, we coupled WWS coding-complete sequence amplification of the CPV genome as a single contig from WW and long-read sequencing.

We detected CPV in all the WW samples screened. Specifically, variants of CPV-2a, 2b and 2c were detected alongside a FPV variant 100% similar in the VP2 genomic region to the Johnson snow leopard strain FPV isolated in 1964 [14] and present in the Felocell vaccine. Our findings, therefore, demonstrate that WWS coupled with long-range PCR and long-read sequencing is a feasible, faster, cost-effective, highly efficient, population-level surveillance system for parvoviruses.

## 2. Methods

### 2.1. Sample Collection and Processing

Archived WW concentrates were analyzed in this study. The samples were originally collected as part of a SARS-CoV-2 WW surveillance study. WW samples were collected using an automated sampler (Teledyne ISCO, Lincoln, NE, USA) that collected ~100 mL aliquots of influent WW over 24 h to create a composite sample. Approximately 150 mL of the composite WW sample was membrane filtered (450 nm) and the filtrate concentrated to ~1 mL using a 10 kDa centrifugal filter (MilliporeSigma, Burlington, MA, USA). The concentrates were subsequently stored at −80 °C. For this study, we recovered concentrates collected from two sites (total population ~500,000 people located in the greater Phoenix metropolitan area, Maricopa County, AZ, USA) once every month from July 2022 to June 2023. For each month, we made a 400 μL concentrate pool consisting of 200 μL from each of the stored concentrates.

### 2.2. Extraction and Nested Polymerase Chain Reaction (nPCR) Assay

Nucleic acid was extracted from 140 μL of the pooled concentrate using the QIAamp mini kit (QIAGEN, Germantown, MD, USA). The extract was used as a template for a nested PCR (nPCR) assay using primers previously described [15,16] with slight modifications. As previously described [3], the extract was used as a template for the first-round assay, which used the Phusion Plus Green master mix (ThermoFisher Scientific, Waltham, MA, USA) to amplify the coding-complete sequence (~4.5 kb) as a single contig. Subsequently, the first-round amplicon was used as template for the second-round assay, which used the GoTaq Green PCR master mix (Promega, Madison, WI, USA) to amplify a ~340 bp fragment spanning the end of NS1 and the beginning of VP1. Thermal cycling conditions on a Biorad C1000 thermal cycler (BioRad Laboratories, Inc., Hercules, CA, USA) for the first-round assay were 94 °C for 3 min, 40 cycles of 94 °C for 30 s, 55 °C for 30 s, and 68 °C for 6 min, and finally 68 °C for 10 min. For the second-round assay, cycling conditions were 94 °C for 3 min, 35 cycles of 94 °C for 30 s, 55 °C for 30 s, and 60 °C for 30 s, and finally 72 °C for 10 min. Subsequently, 5 μL of the second-round PCR product was subjected to electrophoresis on a 2% agarose gel (RPI, Mount Prospect, IL, USA) stained with GelRed (Biotium, Fremont, CA, USA).

### 2.3. Sequencing

For samples positive for the nested assay, the amplicons from the first-round assay were cleaned, pooled and sequenced on a Flongle flow cell (Oxford Nanopore Technologies, San Francisco, CA, USA). Library preparation was performed using the SQK-LSK114 ligation sequencing kit (Oxford Nanopore Technologies, San Francisco, CA, USA) according to the manufacturer’s instructions. Base calling was performed using Guppy as implemented in MinKNOW v24.02.8. The FASTQ reads were trimmed using Porechop v0.2.4, reads between 4400 nt and 4900 nt were selected and assembled using Flye v2.9.1 and subsequently polished using Medaka v1.7.2 as implemented in Nanogalaxy [17].

### 2.4. Phylogenetics, Recombination Analysis, B-Cell Epitope and Structure Prediction

The contigs were used as a query in a BLASTn search of the GenBank database and the top 50 hits for each of the contigs were recovered and assembled into a local database alongside the contigs generated in this study. The database was deduplicated (afterwards referred to as database 1) and subsequently subjected to multiple sequence alignment using MAFFT as implemented in the online server [18]. Afterwards, the alignment was used to infer maximum-likelihood trees in IQ-Tree v1.6.12 [19] after the best model was selected using ModelFinder [20] as implemented in IQ-Tree v1.6.12. It was also subjected to recombination analysis using RDP v5.43 [21]. Since in many CPV studies, researchers sequence many VP2 but few coding-complete sequences, we decided to make database 2, which followed the same workflow as in database 1, except that only the VP2 portion of the contigs sequenced in this study was used to query the GenBank database. Similarity analysis was performed using SDT v1.2 [22]. B-cell epitopes in VP2 were predicted using Bepipred Linear Epitope Prediction 2.0 [23]. The structures of VP2 and NS1 were predicted using AlphaFold 3 [24] and annotated using UCSF ChimeraX v1.8 [25]. Except where stated, software was used with default parameters.

## 3. Results

All twelve (12) concentrates analyzed were positive for the 340 bp second-round amplicon, suggestive of CPV. Consequently, all first-round amplicons were sequenced. In all, 124,261 reads were generated. *De novo* assembly showed 12 contigs (length: 4555 nt to 4675 nt) that typed as parvoviruses (Table 2) and were assembled from 86,858 trimmed and size-selected reads (length: 4400 nt–4900 nt). A Blastn search of the GenBank database typed eleven (11) of the twelve (12) contigs as CPV (GC% = 36%), while the twelfth was typed as FPV (GC% = 35%). This was confirmed by antigenic typing using amino acid residues in antigenic positions in the VP2 protein (Table 2). The eleven (11) CPVs fell into three antigenic groups (2a, 2b and 2c) based on VP2 typing. Eight (8), two (2) and one (1) typed as 2c, 2b and 2a, respectively (Table 2).

**Figure 1:**
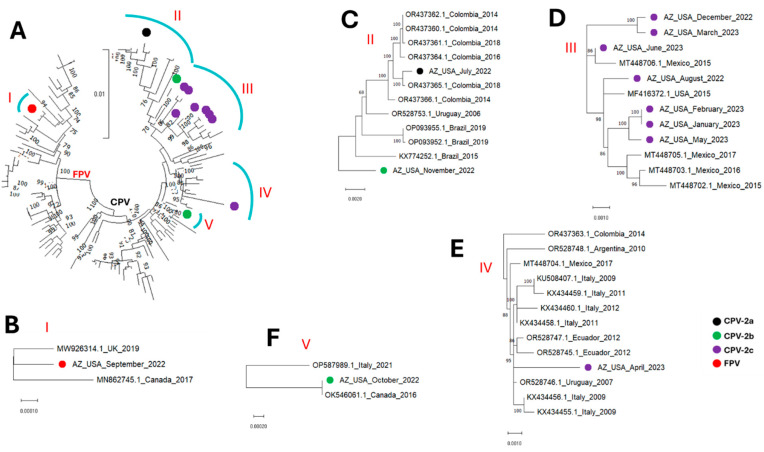
Coding-complete sequence maximum-likelihood tree (model HKY+F+I+G4) of (**A**) the sequences detected in this study alongside those from GenBank (database 1). (**B**–**F**) show zoomed in and leaf annotations of clusters I to V. The sequences detected in this study are highlighted with colored circles.

**Figure 2:**
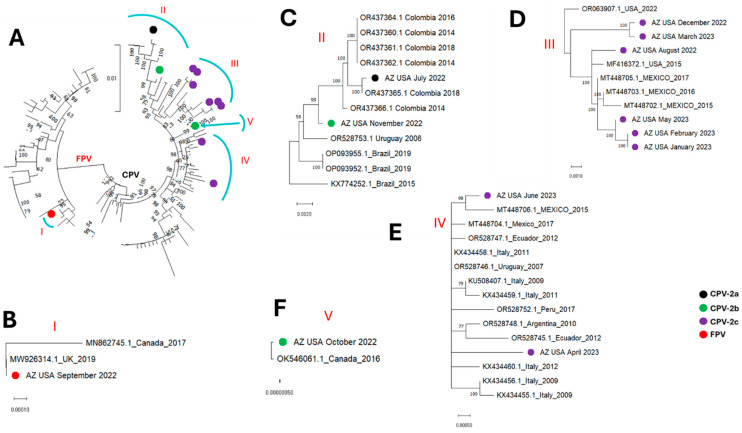
VP2 sequence maximum-likelihood tree (model HKY+F+I+G4) of (**A**) the sequences detected in this study alongside those from GenBank (database 1). (**B**–**F**) show zoomed in and leaf annotations of clusters I to V. The sequences detected in this study are highlighted with colored circles.

**Figure 3:**
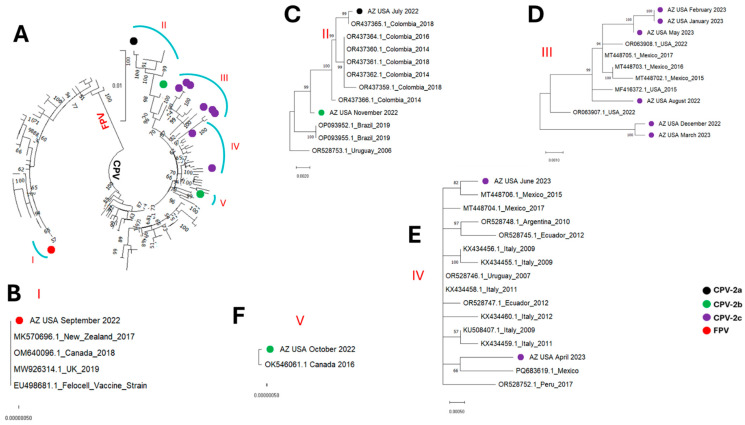
VP2 sequence maximum-likelihood tree (model HKY+F+I) of (**A**) the sequences detected in this study alongside those from GenBank (database 2). (**B**–**F**) show zoomed in and leaf annotations of clusters I to V. The sequences detected in this study are highlighted with colored circles.

**Figure 4:**
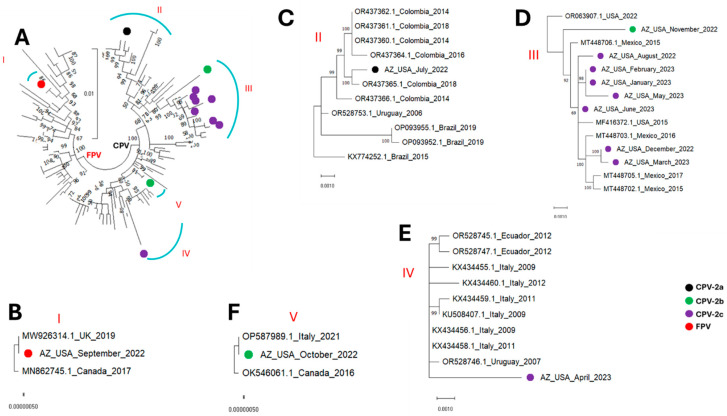
NS1 sequence maximum-likelihood tree (model HKY+F+I) of (**A**) the sequences detected in this study alongside those from GenBank (database 1). (**B**–**F**) show zoomed in and leaf annotations of clusters I to V. The sequences detected in this study are highlighted with colored circles.

All the parvovirus contigs detected in this study fell into five (5) clusters, subsequently referred to as clusters I, II, III, IV and V (Figure 1, Figure 2, Figure 3 and Figure 4). The single FPV detected in this study clustered with other FPVs recovered from GenBank and within that group belonged to cluster I. While BLASTn showed the FPV to be most similar to a variant (OR528756) detected in Argentina in 2009 (Table 2), database 1 based phylogenetic analysis using the coding-complete sequences showed it to be most similar to FPVs detected in the United Kingdom in 2019 (MW926314) and Canada in 2017 (MN862745) (Figure 1, Figure 2, and Figure 4) and this was consistent in phylogenetic trees for the coding-complete sequence (Figure 1), VP2 (Figure 2) and NS1 (Figure 4). However, Figure 3 (which was based on database 2) and Table 3 showed that the FPV detected in this study, the variant detected in the United Kingdom in 2019 (MW926314) and that from New Zealand in 2017 (MK570696) were all of vaccine origin. Specifically, all clusters with the Johnson snow leopard strain FPV isolated in 1964 and present in the Felocell vaccine.

All the CPVs detected in this study clustered with other CPVs recovered from GenBank and belonged to cluster II, III, IV and V (Figure 1, Figure 2, Figure 3 and Figure 4). The only CPV-2a detected in this study was recovered from WW in July 2022 and belonged to cluster II in all four phylogenetic trees (Figure 1, Figure 2, Figure 3 and Figure 4). It clustered with CPVs detected in South American countries (Colombia, Brazil and Uruguay) between 2014 and 2019 (Figure 1, Figure 2, Figure 3 and Figure 4 and Figure S1).

In this study, two CPV-2b variants were detected in wastewater samples collected in October and November 2022. Although recovered in two consecutive months, both variants were phylogenetically distinct. Specifically, in phylogenetic trees for coding-complete sequence and VP2, the October and November 2022 CPV-2b variants belonged to clusters V and II, respectively (Figure 1, Figure 2 and Figure 3). However, while the October 2022 variant remained in cluster V in the NS1 region, the November 2022 CPV-2b variant did not cluster with cluster II in the NS1 region. Rather, it clustered with cluster III variants (Figure 4). In the phylogenetic trees, the October 2022 variant (cluster V) clustered with variants detected in Canada in 2016 (OK546061) and Italy in 2021 (OP587989). Interestingly, the October 2022 variant (cluster V) clustered more closely with the Canada 2016 (OK546061) variant in the coding-complete sequence and VP2 trees (Figure 1, Figure 2 and Figure 3) but the Italy 2021 (OP587989) variant in the NS1 tree (Figure 4). The November 2022 variant clustered with variants detected in South American countries (Colombia, Brazil and Uruguay) in the coding-complete sequence and VP2 (Figure 1, Figure 2 and Figure 3 and Figure S1). However, it clustered with variants detected in North American countries (USA and Mexico) in the NS1 (Figure 4 and Figure S1). Though some phylogeny incongruencies were detected with the CPV-2bs, recombination analysis using RDP5 did not detect any evidence of recombination.

Eight CPV-2c variants were detected in this study. In all four (coding-complete sequence, VP2 and NS1) trees, six variants (August 2022, December 2022, January 2023, February 2023, March 2023 and May 2023) belonged to cluster III and one variant (April 2023) belonged to cluster IV. The eighth variant detected in June 2023 belonged to cluster III in the coding-complete sequence and NS1 tree but to cluster IV in the VP2 trees (Figure 1, Figure 2, Figure 3 and Figure 4). All variants in cluster III were from North America (USA and Mexico), while variants in cluster IV were from North America (Mexico), South America (Ecuador, Argentina and Peru) and Europe (Italy).

**Figure 5:**
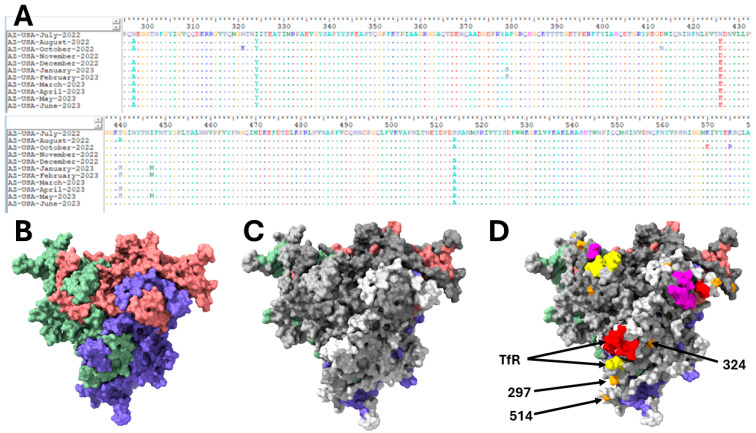
Panel (**A**) shows, in an alignment, regions with amino acid substitutions (outside those detailed in Table 2 except residue 426) in the VP2 of CPVs detected in this study. Panel (**B**) shows the predicted structure of VP2 trimer, showing the three-fold axis of symmetry. Panel (**C**) shows the predicted B-cell epitopes layered on the structure in Panel (**B**). The predicted epitopes are colored gray, light gray and dark gray, respectively, for each monomer. Panel (**D**) shows the footprint of the Transferrin receptor (TfR) (red, yellow and magenta for each of the monomers) [26] layered on the structure in Panel (**C**). Amino acid substitutions (N297A, I324Y and S514A) are also shown in orange. The TfR footprint (spans two VP2 monomers) and amino acid residues are labelled with black arrows in one of the three faces of the three-fold axis of symmetry shown.

Besides the amino acid substitutions in antigenic regions detailed in Table 1 and Table 2, there were three amino acid substitutions (N297A, I324Y, S514A) in the capsid region present in over 50% of the variants detected in this study (Figure 5). Mapped onto the predicted CPV capsid trimmer, we show that these three amino acid substitutions are outside of the Transferrin receptor (TfR) receptor footprint as previously detailed [26] (Figure 5) but are all within the predicted B-cell epitopes.

**Figure 6:**
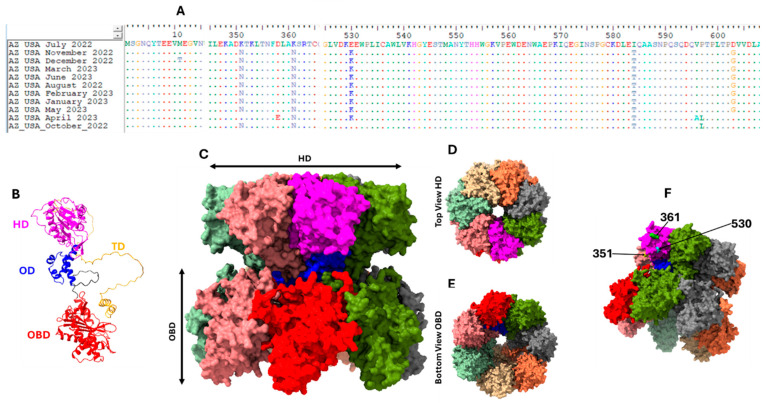
Panel (**A**) shows, in an alignment, regions with amino acid substitutions in the NS1 of CPVs detected in this study. Panel (**B**) shows the predicted structure of NS1 monomer with the four domains (Origin binding domain [OBD], Oligomerization domain [OD], Helicase domain [HD] and transactivation domain [TD]) color-annotated [27]. Panels (**C**–**F**) show the structure of a heptamer [27] with one of the monomers color-annotated (as in Panel (**B**)) to show the domains as color-annotated in Panel (**B**). The remaining monomers have different colors to show them distinctively. Panel (**C**) shows the side view, (**D**) shows the top view from the HD, (**E**) shows the bottom view from the OBD, and (**F**) shows (pointed to by black arrows) the location of amino acid substitutions in the HD found in contigs detected in this study and shown in the alignment in Panel (**A**).

In the NS1 coding region, there were five amino acid substitutions (K351N, K361N, E530K, I584T and D603G) present in over 50% of the variants detected in this study (Figure 6A). Mapped onto the predicted CPV NS1 Heptamer, we show that three (K351N, K361N and E530K,) of these amino acid substitutions map onto the edges of a protrusion on the outer surface of the helicase domain (Figure 6B–F). The remaining two amino acid substitutions (I584T and D603G) are positioned within the transactivation domain (Figure 6A,B).

## 4. Discussion

In this study, we detected both FPV and CPV in municipal wastewater from a population of about 500,000 people in Arizona, USA (Table 2), but we cannot confirm the host(s) species that shed the viruses detected in this study. However, considering both FPV and CPV infect felids [1] and the FPV detected in this study seems to be of vaccine origin, it is possible that the viruses we detected in this study might be from domestic cat feces. It is not clear how these viruses got into the municipal WW tested in this study. However, multiple potential sources and explanations may be hypothesized. First, pet owners in this community flush domestic cat feces into the sewer system (personal communication). Hence, this might likely account for finding domestic cat-infecting viruses in municipal WW in this community. Secondly, the community has over twenty (20) animal shelters and grooming facilities, and cleaning water from these facilities can serve as a potential source of these viruses into the WW system. Thirdly, some pet owners train their domestic cats and dogs to use indoor toilets in houses. This is another source that might account for us finding both CPV and FPV in municipal WW in this population and might suggest that some of the viruses detected here could also be from domestic dog feces. Though unlikely, it is also possible that these viruses could have entered the WW system through surface runoff. Specifically, many pet owners walk their dogs out to the roadside to defecate and afterwards will pick up the feces in plastic fecal bags and dump them in the trash, which is then collected routinely by the city and discarded at the landfill. However, some pet owners do not pick up after their domestic dogs. In addition, there are stray cats in the city, along with foxes, coyotes and raccoons, all in search of food and sometimes being fed by residents. Consequently, animal feces can sometimes be seen by the roadside. Being a desert environment, there is significant effort devoted to efficient water management. Hence, while domestic WW is plumbed to the WW treatment plant, surface runoff is channeled to underground collection basins. Of course, leakages are not impossible and can result in surface runoff contaminating the domestic wastewater, and there are sites like lift stations where this might be more likely.

The FPV variant we detected in this study is 100% identical in the VP2 region with the 1964 Johnson snow leopard strain FPV [14] present in the Felocell vaccine (Figure 3 and Table 3). Considering the evolutionary rate of parvoviruses, it is almost impossible to find two independent isolates detected about 60 years (1964 to 2022) apart being 100% similar in the VP2 region (1744 nt). It is therefore likely that the FPV detected in this study might have come from a recently vaccinated kitten, litter or kindle, although other explanations (like persistence in the environment and improper disposal of unused vaccine) cannot be ruled out without additional epidemiological information. Studies have documented viral DNA detection in the feces of canines and felines for days after vaccination using the modified-live virus vaccines of CPV and FPV [28,29,30]. However, while detection of vaccine-origin virus in WW is quite common for a vaccine like the oral polio vaccine [31], based on sequence data publicly available in GenBank, to our knowledge, this might be the first detection of an FPV vaccine-origin virus in WW. While this might attest to the ability of this vaccine to facilitate secondary vaccination and thereby amplify the impact of administered vaccine doses, it might also be a source of concern. Multiple studies suggest that recombination might be contributing to the evolution of parvovirus genomes [16,32,33]. Consequently, we wonder whether, as with vaccine-derived polioviruses [34,35], there might be vaccine-derived FPVs and/or CPVs with close to wild-type transmissibility and pathogenicity, present and circulating in the population. More studies might be required to explore this possibility and investigate if and/or how such variants might be contributing to the ecology and evolution of parvoviruses. Furthermore, while beyond the scope of this present study, predicting the pathogenicity of CPV and FPV from genomic sequences might be an important future direction. However, establishing such predictive capacity will require experimental studies that will exhaustively map attenuating mutations in vaccine strains and investigate the molecular mechanisms by which these mutations reduce virulence. Coupling such experimental findings with comparative genomics and structural analyses could eventually facilitate reliable prediction of pathogenicity directly from sequence data.

In this study, over the course of 12 calendar months, we detected CPV-2a, 2b and 2c from WW in the same community. The only CPV-2a variant detected was most similar to variants detected in South America (Colombia, Uruguay and Brazil) between 2014 and 2019 and especially to a variant detected in Colombia in 2018 (Figure 1, Figure 2, Figure 3 and Figure 4 and Figure S1). The two CPV-2b variants detected were phylogenetically distinct and seemed to be independent introductions into the population (Figure 1, Figure 2, Figure 3 and Figure 4). We note that while recombination is quite common in CPVs and our data showed phylogeny incongruence in the CPV-2bs, it is possible that the reason RDP5 found no evidence of recombination might be because our algorithm for sequence selection (top 50 hits) might have limited our sample set, which may reduce the power to detect such events. Hence, future studies might benefit from utilizing a more robust sample size to ensure such events are better accounted for. The CPV-2c variants seem to be a mix of variants circulating in the population. From December 2022 to June 2023, only CPV-2c variants were detected. Phylogenetic analysis, however, showed that although all the CPV-2c variants (excluding the April 2023 variant) belong to cluster III, they might not be part of one transmission chain (Figure 1, Figure 2, Figure 3 and Figure 4). Hence, although additional epidemiological data might be needed for confirmation, our findings are consistent with the likelihood that multiple CPV-2c transmission chains were present in the population during the study period. However, that pattern of CPV-2c outcompeting other variants has been previously documented, with studies across Asia, North America, Africa, and South America showing the tendency of CPV-2c to replace other variants once introduced [36].

Irrespective of the cluster, except for variants from Italy, it seems the CPV variants detected in the USA in this study were most similar to variants circulating in North and South America (Figure 1, Figure 2, Figure 3 and Figure 4 and Figure S1). The clustering of the variants detected in this study with variants detected in the Americas suggests they are part of the CPV population circulating in the region. Furthermore, since CPVs are not transmitted in isolation but as part of the virome of the host, our findings suggest that systems might be in place that facilitate or encourage the movement of animal viruses (and possibly other pathogens) across the Americas and hint that some degree of regional confinement might also be at play here (Figure S1). It also suggests that the transboundary transmission of animal viruses in the Americas might be rampant. Hence, there is a need for enhanced, low-cost and efficient early-warning pathogen tracking systems like the one described in this study.

We predicted the structure of the three-fold axis of symmetry on the CPV capsid and layered the predicted B-cell epitopes and TfR footprint [26] on it. Our results showed that the TfR footprint is within the predicted B-cell epitopes (Figure 5). This is consistent with previous experimental findings [2,37] showing that the TfR footprint overlaps footprints of two CPV neutralizing antibodies. Besides the VP2 antigenic sites used for CPV typing (Table 1 and Table 2), we found three more amino acid substitutions (N297A, I324Y and S514A) that were present in more than 50% of the contigs described here. Our results show that these three amino acid substitutions are outside of the TfR receptor footprint detailed in Lee et al. [26] (Figure 5) but are all within the predicted B-cell epitopes. This suggests that amino acid substitutions at these three positions might be driven by host immunological responses, and the literature [2,38] shows experimental evidence supporting our findings. Specifically, Hartmann et al. [38] described two sites (A and B) on the CPV capsid that are footprints for two CPV neutralizing antibodies and residues 297 and 514 fall within the site B neutralizing antibody footprint. Furthermore, residue 324 falls within the footprint of a single-chain variable fragment (scFv) described by Adu et al. [2]. It is important to note that CPV variants with amino acid substitutions at positions 297 and 324 have been described in Europe and Asia [39,40].

The NS1 protein has been shown to be a multidomain protein with the ability to form oligomers and perform various functions, including inducing apoptosis, type I interferon responses, DNA damage responses, DNA binding and recruitment of transcription factors in a bid to enhance promoter activity [27]. In this study, there were five amino acid substitutions (K351N, K361N, E530K, I584T and D603G) present in over 50% of the NS1 variants detected (Figure 6A). I584T and D603G seem to be within the transactivation domain (Figure 6A,B). However, K351N, K361N and E530K mapped onto the edges of a protrusion on the outer surface of the helicase domain (Figure 6F). It is not clear if and/or how these substitutions might impact the function of NS1. However, the position of K351N, K361N and E530K and their clustering (Figure 6F) suggest that these substitutions might be part of the footprint of one or more proteins that bind to the NS1 protein as it performs one or more of its plethora of functions. Future experimental exploration of these amino acid substitutions might be necessary to fully understand their impact on NS1 function.

The findings of this study show that WW surveillance of CPV and FPV is feasible. Monitoring the diversity of these viruses in WW poses a unique challenge because typing requires monitoring coevolution across a genomic region spanning ~1500 nt (~500 aa) (Table 1 and Table 2). This necessitated the coupling of long-range PCR with long-read sequencing. In this study, we confirm the feasibility of coupling WW surveillance, coding-complete sequence amplification of the parvovirus genome as a single contig from WW and long-read sequencing for exploring their diversity on a population scale. As of the time of writing, we are not aware of any published study utilizing this strategy to monitor parvovirus diversity in communities. This approach provides a cost-effective solution to parvovirus surveillance by facilitating insights into pathogen diversity by monitoring thousands of canines and felines in a city at a population scale, thereby constituting an early warning system. Furthermore, this approach does not rely on samples collected in clinics and consequently provides insight into virus diversity in the population (including asymptomatic infections) that might not be reaching clinics. As in our previous study [3], here we used a modified version of the coding-complete sequence amplification assay previously described [15,16]. Specifically, we amplified the coding-complete sequence as a single contig. While amplifying the genome in two or more overlapping fragments works well for clinical samples, for WW it might not be ideal because samples might be coming from multiple sources and consequently contain multiple variants. In addition, the single contig approach allows us to confidently explore coevolving sites in both the structural and nonstructural genomic regions from contigs recovered from WW without concerns that we might be dealing with artificial chimeras.

The results of this study show that parvovirus (including CPV and FPV) surveillance can be performed in communities using WW to complement clinical case surveillance. Coupled with coding-complete sequence amplification as a single contig from WW and long-read sequencing, this can facilitate the availability of genomic information on viruses from asymptomatic and/or subclinical cases. Such information will be valuable for molecular epidemiology, vaccine development, update of vaccine strains and monitoring the contribution of vaccine origin variants to parvovirus ecology and evolution. This approach can be expanded to monitor other parvoviruses that infect humans, like parvovirus B19 and human bocavirus and other DNA viruses of medical importance to humans like human papilloma virus and hepatitis B virus. We have also shown the utility of this approach for RNA viruses [9]. Finally, WW represents a unique One Health virus surveillance matrix because it combines virus variants from humans, domestic animals, wildlife, and the shared environment. Our detection of CPV/FPV variants in WW highlights how virus diversity and abundance in animals and in the environment can be monitored alongside human viruses within the same system and adds to the body of evidence supporting the utility of municipal WW surveillance for a One Health approach to pathogen surveillance and, consequently, pandemic preparedness.

## Figures and Tables

**Table 1:** Antigenic variants at major antigenic sites in VP2 are shown as detailed in Miranda and Thompson, 2016 [1]. Changes are highlighted in red.

	Amino Acid Residue at Major Antigenic Sites in VP2											
Virus Type	80	87	93	101	103	300	305	323	426	555	564	568
FPV	K	M	K	I	V	A	D	D	N	V	N	A
CPV-2	**R**	M	**N**	I	**A**	A	D	**N**	N	V	**S**	**G**
CPV-2a	R	**L**	N	**T**	A	**G**	**Y**	N	N	**I**	S	G
CPV-2b	R	L	N	T	A	G	Y	N	**D**	**V**	S	G
CPV-2c	R	L	N	T	A	G	Y	N	**E**	V	S	G

**Table 2:** Details of parvovirus contigs generated in this study and their VP2 antigenic type. Changes from 2a are highlighted in red. # means number and nt means nucleotides.

					Amino Acid in Select Positions in VP2												
Name	# Reads	# Size-Selected Reads Length [4.4 K–4.9 K]	Contig Length (GC%)	Closest GenBank Hit (% Similarity, Year, Country)	80	87	93	101	103	300	305	323	426	555	564	568	Type
AZ-USA-July-2022	20,334	16,183	4610 nt (36%)	OR437365.1, (99.85%, 2018, Colombia,)	R	L	N	T	A	G	Y	N	N	V	S	G	CPV-2a
AZ-USA-August-2022	14,583	12,467	4574 nt (36%)	MF416372.1 (99.71%, 2015, USA)	R	L	N	T	A	G	Y	N	E	V	S	G	CPV-2c
AZ-USA-September-2022	8000	6786	4555 nt (35%)	OR528756.1 (99.21%, 2009, Argentina)	K	M	K	T	V	A	D	D	N	V	N	A	FPV
AZ-USA-October-2022	13,940	9828	4579 nt (36%)	OP587989.1 (99.74%, 2021, Italy)	R	L	N	T	A	G	Y	N	D	V	S	G	CPV-2b
AZ-USA-November-2022	11,247	9260	4675 nt (36%)	KX434458.1 (99.36%, 2011, Italy)	R	L	N	T	A	G	Y	N	D	V	S	G	CPV-2b
AZ-USA-December-2022	11,743	8353	4566 nt (36%)	MT448706.1 (99.47%, 2015, Mexico)	R	L	N	T	A	G	Y	N	E	V	S	G	CPV-2c
AZ-USA-January-2023	9167	7369	4673 nt (36%)	MF416372.1 (99.67%, 2015, USA)	R	L	N	T	A	G	Y	N	E	V	S	G	CPV-2c
AZ-USA-February-2023	13,072	3346	4626 nt (36%)	MF416372.1 (99.67%, 2015, USA)	R	L	N	T	A	G	Y	N	E	V	S	G	CPV-2c
AZ-USA-March-2023	4298	2040	4656 nt (36%)	MT448706.1 (99.47%, 2015, Mexico)	R	L	N	T	A	G	Y	N	E	V	S	G	CPV-2c
AZ-USA-April-2023	5941	3186	4574 nt (36%)	KX434458.1 (99.54%, 2011, Italy)	R	L	N	T	A	G	Y	N	E	V	S	G	CPV-2c
AZ-USA-May-2023	6741	4892	4622 nt (36%)	MF416372.1 (99.67%, 2015, USA)	R	L	N	T	A	G	Y	N	E	V	S	G	CPV-2c
AZ-USA-June-2023	5195	3148	4663 nt (36%)	MT448706.1 (99.87%, 2015, Mexico)	R	L	N	T	A	G	Y	N	E	V	S	G	CPV-2c
Total	124,261	86,858															

**Table 3:** Similarity of the VP2 complete coding sequences in Figure 3B (cluster 1) showing all are most likely vaccine-origin FPV strains. Similarity analysis was performed using SDT.

First Sequence	Second Sequence	Similarity (%)
EU498681.1_Felocell_Vaccine_Strain	MK570696.1_New_Zealand_2017	100
EU498681.1_Felocell_Vaccine_Strain	AZ_USA_September_2022	100
EU498681.1_Felocell_Vaccine_Strain	MW926314.1_UK_2019	100
EU498681.1_Felocell_Vaccine_Strain	OM640096.1_Canada_2018	100
AZ_USA_September_2022	OM640096.1_Canada_2018	100
MW926314.1_UK_2019	AZ_USA_September_2022	100
MK570696.1_New_Zealand_2017	OM640096.1_Canada_2018	100
MW926314.1_UK_2019	MK570696.1_New_Zealand_2017	100
MW926314.1_UK_2019	OM640096.1_Canada_2018	100
MK570696.1_New_Zealand_2017	AZ_USA_September_2022	100

## Data Availability

The genomes described in this study have been deposited in the GenBank under accession numbers PV137620-PV137631.

## Supplementary Materials

The following supporting information can be downloaded at: https://www.mdpi.com/article/10.3390/microorganisms13092124/s1, Figure S1: Global source (excluding USA) of CPVs and FPV belonging to the five clusters detected in USA wastewater in this study. Countries represented in more than one cluster are indicated with a black star. Note that variants from the UK and New Zealand are vaccine origin. Table S1: Accession numbers of sequences retrieved from GenBank for use in this study.
