# Supplementary material for: Canine Parvovirus and Vaccine-Origin Feline Panleukopenia Virus in Wastewater, Arizona, USA: July 2022–June 2023
Source: Microorganisms. 2025 Sep 11;13(9):2124. doi: 10.3390/microorganisms13092124 (PMC12472840; doi:10.3390/microorganisms13092124)
Supplement: Supplementary file 1 [file microorganisms-13-02124-s001.zip › microorganisms-3815540-supplementary.pdf]

Table S1: Accession numbers of sequences retrieved from GenBank for use in this study.

S/N	Accession #	S/N	Accession #	S/N	Accession #	S/N	Accession #	S/N	Accession #
1	OR437365.1	31	OM640101.1	61	OP587982.1	91	MN419005.1	121	MW926314.1
2	OR437366.1	32	OR528749.1	62	OP587986.1	92	OQ815873.1	122	MN862745.1
3	OR437364.1	33	OR437363.1	63	OP587983.1	93	EU498714.1	123	U22191.1
4	OR437362.1	34	MF423125.1	64	OM640102.1	94	OP985517.1	124	M24004.1
5	OR437361.1	35	EF011664.1	65	OM640099.1	95	OP985515.1	125	MN127781.1
6	OR437360.1	36	OP588011.1	66	M24000.1	96	OP985511.1	126	D00765.1
7	OR437359.1	37	OP587967.1	67	OK546060.1	97	PQ880169.1	127	MH165482.1
8	OP093952.1	38	OP587964.1	68	MZ476774.1	98	MF541125.1	128	MH165481.1
9	OP093955.1	39	OP587965.1	69	OR063907.1	99	AB000056.1	129	MF069446.1
10	OR528753.1	40	OP588014.1	70	MW365734.1	100	PQ807638.1	130	MN862743.1
11	MT448703.1	41	OP588007.1	71	JN867610.1	101	MW847201.1	131	MZ508522.1
12	MT448705.1	42	OP587970.1	72	JN867611.1	102	MW847159.1	132	M24002.1
13	OR063908.1	43	OP587975.1	73	OR063909.1	103	EU498717.1	133	M24005.1
14	MF416372.1	44	JQ268283.1	74	JN867614.1	104	OQ398418.1		
15	MT448702.1	45	OM640097.1	75	OK546061.1	105	OQ398393.1		
16	OR528746.1	46	MG434745.1	76	MH476588.1	106	OQ398392.1		
17	KX434458.1	47	MG434743.1	77	MH106699.1	107	OQ398391.1		
18	OR528747.1	48	MG434738.1	78	MH106700.1	108	OQ398390.1		
19	MT448704.1	49	MG434740.1	79	MF805794.1	109	OQ398389.1		
20	KU508407.1	50	MG434739.1	80	MF805798.1	110	OQ398388.1		
21	KX434459.1	51	OP588012.1	81	MT165692.1	111	OQ398387.1		
22	MT448706.1	52	OP587976.1	82	OR528751.1	112	OQ398386.1		
23	OR528748.1	53	OP587979.1	83	OR528750.1	113	MK570696.1		
24	OR528752.1	54	OP587981.1	84	OP587989.1	114	MK570703.1		
25	KX434460.1	55	OP588013.1	85	OP595739.1	115	MK570676.1		
26	KX434456.1	56	MN862741.1	86	EU498681.1	116	MK570701.1		
27	KX434455.1	57	MF069442.1	87	EU498680.1	117	EU659112.1		
28	OR528745.1	58	KX434454.1	88	D88287.1	118	X55115.1		
29	PQ683619.1	59	OP587988.1	89	ON605652.1	119	U22187.1		
30	OK546062.1	60	KX434457.1	90	OQ615264.1	120	OM640096.1		

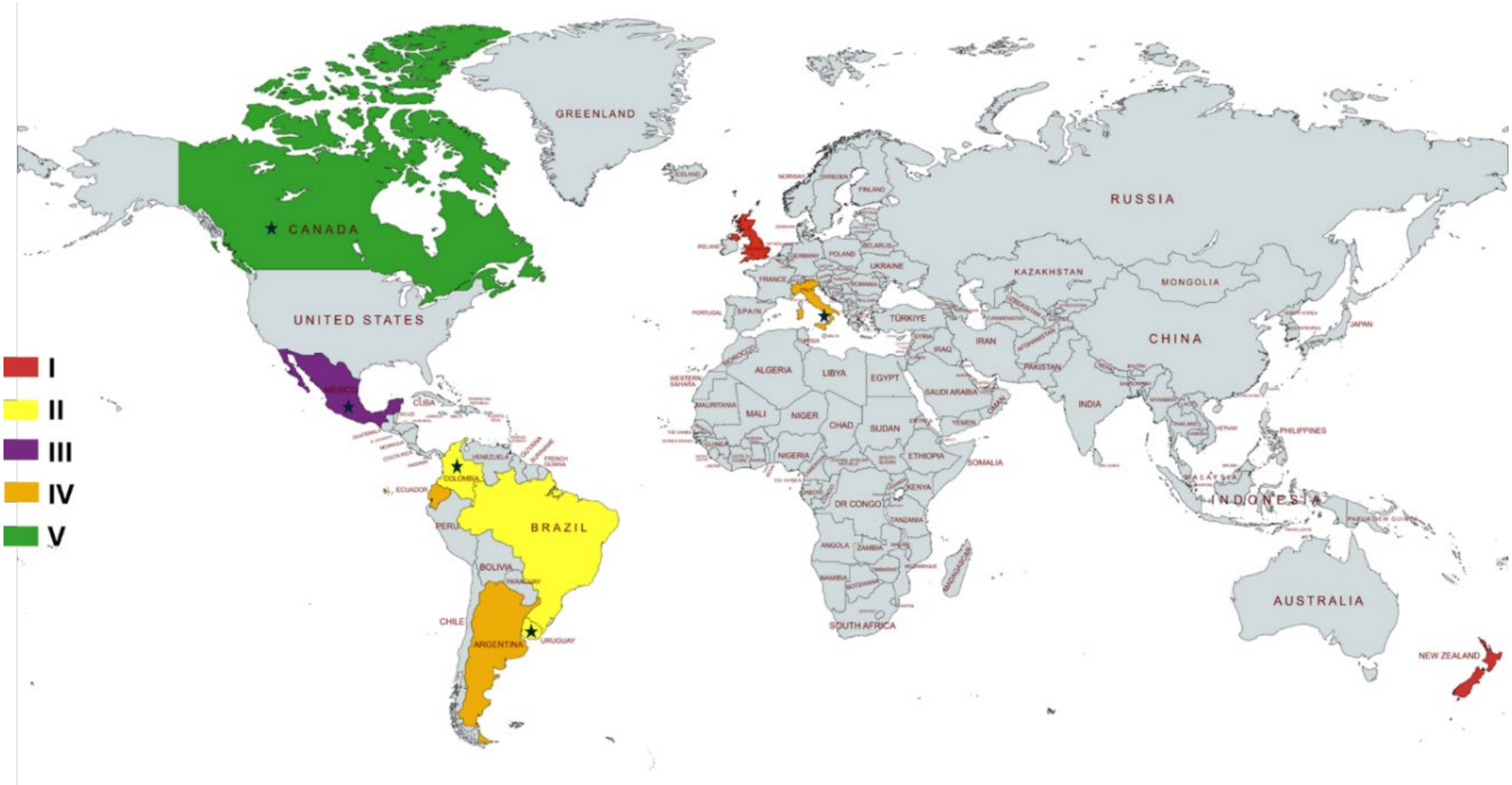

Figure S1. Global source (excluding USA) of CPVs and FPV belonging to the five clusters detected in USA wastewater in this study. Countries represented in more than one cluster are indicated with a black star. Note that variants from the UK and New Zealand are vaccine origin.
